# Experience and perceived quality of care of patients and their attendants in a specialized mental hospital in Bangladesh

**DOI:** 10.1186/s13033-019-0303-x

**Published:** 2019-06-22

**Authors:** Nazmun Nahar Nuri, Malabika Sarker, Helal Uddin Ahmed, Mohammad Didar Hossain, Claudia Beiersmann, Albrecht Jahn

**Affiliations:** 1 Institute of Public Health, Ruprecht-Karls-Universität Heidelberg, INF 130/3, 69120 Heidelberg, Germany; 2 James P. Grant School of Public Health, BRAC University, 66 Mohakhali, Dhaka, 1212 Bangladesh; 3 National Institute of Mental Health, Sher-e-Bangla Nagar, Dhaka, 1200 Bangladesh; 4 Foundation for Advancement of Innovations in Technology and Health, 1/15A Iqbal Road, Dhaka, 1207 Bangladesh

**Keywords:** Quality of care, Patient satisfaction, Mental health, Specialized hospital, Bangladesh

## Abstract

**Background:**

A person’s perception of health service quality depends on his or her expectations and priorities. If the perception of care is good, then it eventually enhances future health service utilization, adherence to treatment and desired outcomes. Understanding a patient’s perspective is key for delivering a better quality patient-centred health care service. This study explored experience and perception of patients and their attendants of mental health care services at the National Institute of Mental Health (NIMH) which is the only national level mental health institute in Bangladesh. NIMH is located in the capital city and provides specialized mental health care services for the whole population.

**Methods:**

A facility-based cross-sectional study was conducted using a mixed-method design at the NIMH. A total of 40 respondents (patients, or their attendants if the patient was minor or unable to respond due to lack of mental stability) visiting the outpatient department (OPD) of NIMH were selected by purposive sampling. For each of the ten ICD 10 categories (10th revision of International Classification of Diseases by the World Health Organization [WHO]) for mental disorders, four patients were chosen. Finally, 13 patients and 27 attendants (on behalf of 17 minor patients and 10 adult patients unable to respond) participated in the interview.

**Results:**

The respondents rated 34 short statements clustered around four dimensions of care (accessibility, interpersonal communications, condition of the waiting and consultation rooms, and general quality of OPD services) and we interpreted those scores as follows: 7.6–10 very satisfied/very good quality, 5.1–7.5 satisfied/good quality, 2.6–5.0 dissatisfied/poor quality and 1.0–2.5 completely dissatisfied/very poor quality. For accessibility and interpersonal communications, the patients perceived care as very good (average scores on a Likert scale of 1–10 were 8.3 and 7.6, respectively). The respondents considered the condition of the waiting and consultation rooms and general quality of OPD care as good (average scores 5.8 and 7.1, respectively). NIMH had serious lack of resources in terms of functional medical equipment and physicians appointed, which negatively impacted on the service quality.

**Conclusions:**

Patients receiving services from the NIMH OPD had a positive perception of the quality of care in general. But, at an individual level, some respondents expressed dissatisfaction. Our findings are informative for quality improvement and client-oriented care service planning at NIMH, Bangladesh.

## Background

Good quality care is defined as “providing patients with appropriate services in a technically competent manner, with good communication, shared decision making, and cultural sensitivity” [1]. Health service quality is a subjective, complex and multi-dimensional concept. It comprises tangible (e.g. hygiene) and intangible (e.g. empathy) aspects [2]. A person considers a service to have good or bad qualities depending on his or her own expectations and priorities, which might differ from technical quality [2]. Patient satisfaction is significantly influenced by financial affordability particularly in low resource setting [3]. Therefore, it is possible that a patient is satisfied with care of low technical quality, but can be dissatisfied with care of good technical quality [2].

Although technical aspects of care are considered to be important by patients, their opinions about health service quality are generally based on their assessment of non-clinical aspects of care, e.g. availability and accessibility, cleanliness, comfort, privacy and a quiet and attractive environment [2]. Accessibility of services, interpersonal relations between the provider and the patient, facility infrastructure and administrative conditions and the technical and social competence of the provider are key elements influencing patient’s perception of the quality of care [2, 4].

The Bangladesh National Health Policy of 2011 has two objectives: (i) expanding the availability of client-centred, equity-focused and high-quality health care services and (ii) motivating people to seek care based on their right to health [5]. The national Health and Population Sector Programme of Bangladesh is committed to improving health service quality [6]. While measuring the quality of health care, patient satisfaction is an important and commonly used indicator that influences clinical outcomes and patient retention [7, 8]. Future utilization of psychiatric services depends on how satisfied patients are with the services received [4]. So, by understanding the patients’ perspective, a better quality, patient-centred health care can be delivered and premature termination of therapy can be avoided [9].

Although a large part of the non-communicable disease burden is caused by mental ill-health, it is widely neglected [10]. Mental health systems are under-resourced particularly in low- and middle-income countries [11]. An estimated 90 percent of the mental health patients in low-resource countries go untreated [12]. Lack of infrastructural capacity and other resources like training programs, skilled staff and medication are key factors limiting the effectiveness of mental health care [13]. The recent inclusion of mental health in the Sustainable Development Goals acknowledges this priority in the health sector and global development agendas [14].

The prevalence of mental health problems among Bangladeshi adults is 16.1% [15] and for children 5–10 years old, 15.2% [16]. According to the World Health Organization neuropsychiatric disorders contribute to 11.2% of the total disease burden in Bangladesh [17]. However, only 0.44% of the national health budget was allocated for mental health [17]. Bangladesh not only has a lack of resources, but there is a disproportionate distribution of available resources within the health sector [5].

The quality of health care is poor in both public and private sectors in Bangladesh. There is little assessment of the quality of provider care, low levels of professional knowledge and poor application of skills [5]. Bangladesh does not have a formal body for arbitration of complaints against health providers. Hospital or clinic authorities address complaints and disputes independently, without involving the government or legal entities [5]. In addition, there is no human-rights review body to inspect mental health facilities, nor is there a specific mental health authority [5].

National Institute of Mental Health (NIMH) is the only Bangladeshi national-level mental health institute (and has an academic faculty). NIMH is situated in the capital city, and has a 200-bed specialized mental hospital that caters to the whole country with a population of 161 million [18]. Our study explored experience and perception of patients and their attendants about the quality of care received at the NIMH OPD. To our knowledge, to date, no study has explored Bangladeshi patients’/attendants’ perception and experience of mental health care services. Therefore, we believe that this study’s findings will function as evidences and be quite useful for health service managers at a local level and health service planners at a central level for improving the quality of and access to mental health care services.

## Methods

### Study design and study site

This study was a facility-based cross-sectional study using mixed methods. The data was collected between July and September 2016. Quantitative part included a survey among OPD patients/attendants, and preparation of checklists and analysis of data gathered from yearly health bulletins and OPD registers. Qualitative data included in-depth interviews of OPD patients/attendants. Our study site NIMH is located in Dhaka and plays a vital role in providing specialized mental health care to the whole Bangladeshi population. Since its establishment in 2001, this hospital has provided care to 286,215 patients in OPD, 21,785 in the inpatient department and 16,420 in the emergency department. In 2015 alone, 42,703 patients received services in the NIMH OPD and 2501 patients in the inpatient department [19].

### Study sample

A total of 40 respondents (patients or their attendants) visiting the NIMH OPD were interviewed. At first, four patients were selected in each of the ten ICD 10 categories for mental disorders by purposive sampling. The diagnoses of patients selected for our study was made by the OPD providers during consultation, and the first author spontaneously recruited patients exiting any of the OPD consultation/service rooms. We assumed that data saturation would be achieved at the end of 40 interviews. Various types of mental health problems cause distinct levels of disability and require specific care. So, we decided to include a similar number of respondents from each of the ICD 10 categories for mental disorders to ensure diversity and representativeness. As access to mental health services might vary across age groups and gender [20], we recruited two male and two female patients as well as two adult and two minor patients for each of the ICD 10 categories where possible. Since this was not possible for all ICD 10 categories, we ultimately recruited 23 male and 17 female patients including 23 adult and 17 minor patients [21].

Before conducting interview, the mental stability of the patients selected for the study was determined by the OPD providers in charge. If a patient was not in the stable mental state required for our interview, we interviewed his/her attendant. Thus, in case of ten adult patients an attendant was interviewed instead. In addition to that, for all minor patients (under 18 years of age) an adult attendant was interviewed. In total, 13 patients and 27 attendants (close family member, e.g. spouse, parent, sibling) were interviewed at the end. The inclusion criteria were: a patient/his or her attendant who had had a consultation with a doctor at the NIMH OPD at least once and who agreed to participate in this study [21].

Besides interviewing patients or their attendants, information published in the online health bulletins available from 2012 to 2016 and data from the OPD registers between January and June 2016 were also analysed.

### Study tool

We used a semi-structured in-depth interview guide and a structured questionnaire to collect data. Both were developed in English and then translated into Bengali by the first author, who is a native Bengali speaker. One native Bangladeshi mental health expert and two Bangladeshi public health experts reviewed the translated tools [21]. The focus of the in-depth interviews was to learn in detail about the experience and perception of the respondents regarding care services at NIMH. The focus of the structured questionnaire was to record information on the subjects’ perceived quality of care.

The structured questionnaire was developed with the help of an already existing and validated tool used in a study of Malawian women to rate maternal and new-born care services [4]. The psychometric scale used in that study was based on the theoretical model of Wilde, Starrin, Larsson and Larsson [22], which generated understanding of the perceived quality of care in the light of two conditions: availability of resources at the care organization and the patients’ preferences. We modified that tool to make it suitable for mental health care and the country context. Moreover, we piloted the tool and made necessary changes prior to final data collection. Our scale was constituted to measure four dimensions of quality perception: accessibility, interpersonal communications, condition of the waiting and consultation rooms and general quality of OPD care services.

The composite perception scale to measure perceived quality of care consisted of 34 short statements. Those statements were clustered around four dimensions of care; accessibility (9 statements), interpersonal communications (8 statements), condition of the waiting and consultation rooms (8 statements) and general quality of OPD services (9 statements). The respondents rated the statements on a psychometric Likert-type scale of 1 to 10, whereby 1 was complete disagreement and 10 was complete agreement with the statement.

To record information on the availability and the functionality of medical equipment we used an inventory checklist. Online yearly health bulletins (2012–2016) were analysed to gather further information on medical equipment and the human resource structure at NIMH. We also collected data from OPD registers for the period January–June 2016 to calculate the number of daily consultations done by each physician.

### Data analysis

Quantitative data from the Bengali survey was entered directly into an English format by the first author. English format was also used to record quantitative data from yearly health bulletins and OPD registers, and on the inventory checklist. Quantitative analysis was performed using Stata version 14.

The in-depth interviews conducted in Bengali were transcribed directly into English by the first author. Content analysis was performed for the qualitative data using NVivo 11. Deductive coding was performed based on the quantitative questionnaire and the codes were arranged according to four quality dimensions: accessibility, interpersonal communications, condition of the waiting and consultation rooms, and general quality of OPD services. While reading and re-reading of the transcripts we did not find any new code/theme beyond our deductive codes and four quality dimensions coming out, so did not feel the need for performing inductive coding.

## Results

The findings on the perceived quality of care at NIMH from the quantitative survey and in-depth interview are presented first. Next, findings on medical equipment non-functionality and lack of trained human resources are presented as related to the participants’ perception of care.

### Demographic characteristics of the patients and respondents

Table 1 presents the demographic characteristics of the patients who were initially selected and the respondents (patients, or their attendants if the patient was minor or unable to respond due to lack of mental stability) who were finally interviewed. Of a total of 40 initially selected patients, 23 (57.5%) were adults. The mean age of patients was 25.5 years (range 3–65 years). Slightly more than half (57.5%) of all patients were male, and 52.5% lived in urban area. The majority of the adult patients (69.6%) were unemployed. Slightly more than half of the adult patients (56.5%) were married, living with a spouse, and had a 6th–12th grade education. Just over half (58.8%) of the minor patients were attending school [21]. The number of NIMH visits by our respondents ranged between one and 90 with a median of five.

Of a total of 40 respondents interviewed finally, 13 were patients and 27 were attendants. The mean age of attendants was 42.7 years (range 21–68 years). More than two-third (70.4%) of the attendants were female and all of the attendants were married and living with a spouse. Slightly more than half (51.8%) of the respondents were living in urban area, two-third (66.6%) were unemployed and the biggest group (44.4%) had up to 5th grade education. The majority (70.4) of the attendants were parents of the patients followed by spouse (18.5%).

**Table 1:** Demographic characteristics of the patients initially selected (N = 40) and the respondents finally interviewed (13 patients and 27 attendants)

Variable	Patients initially selected (total 40) N (%)	Respondents interviewed	
		Attendants (total 27) N (%)	Patients (total 13) N (%)
Age			
< 18 years	17 (42.5)	0	0
18 years or above	23 (57.5)	27 (100)	13 (100)
Sex			
Male	23 (57.5)	8 (29.6)	9 (69.2)
Female	17 (42.5)	19 (70.4)	4 (30.8)
Marital status (adults)			
Single	5 (22.2)	0	5 (38.5)
Residing with the spouse	13 (56.5)	27 (100)	6 (46.1)
Abandoned/divorced/widowed	5 (22.2)	0	2 (15.4)
Place of residence			
Urban	21 (52.5)	14 (51.8)	9 (69.2)
Rural	11 (27.5)	8 (29.6)	1 (7.7)
Semi-urban	8 (20)	5 (18.5)	3 (23)
Employment status (adults)			
Currently working	7 (30.4)	9 (33.3)	6 (46.2)
Not working	16 (69.6)	18 (66.6)	7 (53.8)
Educational status			
Among adults			
Up to 5th grade	7 (30.4)	12 (44.4)	3 (23)
6th–12th grade	13 (56.5)	11 (40.7)	7 (53.8)
> 12th grade	3 (13)	4 (14.8)	3 (23)
Among minors (N = 17)			
< 5 years of age	3 (17.6)	–	–
Attending school	10 (58.8)	–	–
Dropped out (after 5th grade)	1 (5.9)	–	–
Never attended school	3 (17.6)	–	–
Relationship of attendants with patients (N = 27)			
Parents	–	19 (70.4)	–
Children	–	1 (3.7)	–
Grandparents	–	1 (3.7)	–
Spouse	–	5 (18.5)	–
Sibling	–	1 (3.7)	–

### Experience and perceived quality of care received at NIMH OPD

Perceived quality of care scores on the Likert scale were interpreted as follows: 1.0–2.5 completely dissatisfied/very poor quality, 2.6–5.0 dissatisfied/poor quality, 5.1–7.5 satisfied/good quality and 7.6–10 very satisfied/very good quality.

Accessibility of OPD care and interpersonal communications were perceived to be very good; (average scores 8.3 and 7.6, respectively). The respondents considered the condition of waiting and consultation rooms and the general quality of OPD care services as good (average scores 5.8 and 7.1, respectively). Table 2 presents the ratings of statements regarding various quality aspects of OPD care in four dimensions.

**Table 2:** Ratings of quality aspects of NIMH OPD care in four dimensions

Sl. nr.	Quality aspects	Minimum score provided	Maximum score provided	Average Score	Standard Deviation	Number of dissatisfactory score (≤ 5)
Care dimension: accessibility (N = 40)						
1	Information and directions on service points	2	10	8.2	2.2	6
2	Availability of OPD staff for consultation tickets	2	10	9.5	1.5	2
3	Information on fees	1	10	8.8	2.3	5
4	Waiting time for services	1	10	7.0	2.8	13
5	Availability of OPD provider for services	2	10	8.5	2.2	5
6	Availability of advised laboratory tests at NIMH	1	10	5.3	3.7	8 (N = 14)
7	Availability of prescribed medications at NIMH dispensary	2	10	8.0	2.4	9
8	Affordability of the costs	6	10	9.3	1.1	0
9	Existence of informal payments	7	10	9.7	0.9	0
Care dimension: interpersonal communications (N = 40)						
10	Being greeted and asked to take a seat	1	10	8.6	2.4	6
11	Gentle/sympathetic behaviour	1	10	8.4	2.4	7
12	Privacy	1	10	7.9	2.7	10
13	Explanation on current health condition	1	10	7.5	3.2	9
14	Encouragement to ask questions	1	10	7.5	2.9	10
15	Response to concerns/questions	1	10	7.4	2.9	11
16	Sufficient consultation time	1	10	6.4	3.2	17
17	Gentle behaviour and helpfulness of other OPD staffs	1	10	7.2	3.1	11
Care dimension: condition of the waiting and consultation rooms (N = 40)						
18	Cleanliness	1	10	6.5	2.6	17
19	Sufficient light and ventilation	3	10	7.8	2.0	6
20	Comfortability	1	10	6.2	2.8	15
21	Availability of seating	1	10	5.8	2.6	20
22	Availability of OPD patients’ toilet	1	10	5.6	3.2	21
23	Cleanliness and usability of toilet	1	10	4.4	2.9	13 (N = 20)
24	Availability of water/toilet paper in toilet	1	10	5.9	3.1	9 (N = 20)
25	Availability of waste bin	1	10	4.4	3.2	28
Care dimension: general quality of OPD care services (N = 40)						
26	Attentive and patient hearing by care provider	1	10	7.7	2.9	9
27	Further query by care provider	1	10	7.1	3.2	13
28	Physical examination/observation	1	10	7.2	3.2	12
29	Explanation about treatment/advice/possible prognosis	1	10	7.0	3.2	14
30	Information on possible side effects of medication	1	10	5.0	3.5	21
31	Information on follow up visit	4	10	8.9	1.8	4
32	Professional competence of care provider	1	10	7.8	2.8	8
33	Instructions by pharmacist about the intake of medication	1	10	5.6	3.5	20
34	Overall satisfaction about the quality of OPD care	2	10	7.9	2.0	5

Some respondents did not have experience in the quality aspect 6, 23 and 24; therefore, they did not rate those statements

#### Accessibility

The perceived quality of accessibility to services was measured by nine statements: (1) availability of information and direction regarding various service points, (2) availability of OPD staff for consultation ticket, (3) information on fees, (4) availability of OPD providers for services, (5) waiting time, (6) availability of advised laboratory tests in the hospital, (7) availability of prescribed medications in the hospital, (8) affordability of the costs and (9) existence of informal payment. The average score for this sub-section was 8.3. Except of waiting time and laboratory tests availability at NIMH all statements in this section were rated as very good with an average score of ≥ 8. All respondents rated the statements about the affordability of the costs and informal payment as satisfactory (≥ 6). The following quote shows a positive view about NIMH:

*“I preferred this hospital because I get the medicaments for free here. I am a student, so have financial constraints.” (23*-*year*-*old male patient with depression)*

For waiting time, the average score was 7.0, although 13 respondents scored this as dissatisfactory (≤ 5). During in-depth interviews, some respondents expressed their unhappiness about long service waiting times. One respondent stated:

*“The services are ok here, but every time I have to wait a long time until I am called inside the doctors’ room. Particularly in this Shishubikash [special OPD clinic for child patients] there are so many patients that there is always a long queue. It is very difficult to wait this long with a sick child.” (Mother of an 11*-*year*-*old male patient with autism)*

The average score for the availability of laboratory tests was 5.3, from 14 respondents (35%) who reported as having had (or the patient having had) a laboratory test as part of their treatment procedure. Of these respondents 8/14 (57%) scored laboratory test availability as dissatisfactory (≤ 5). In the in-depth interviews, partial or complete unavailability of laboratory tests in the hospital were noted. This situation results in increased treatment costs at NIMH and inconvenience. The following quote from one respondent illustrates the difficulties he faced with his laboratory tests:

*“The cost of treatment is very low. I had to spend almost nothing here. But the [laboratory] tests I had to do outside were very expensive. Moreover, I had to look for an alternate diagnosis centre, and had to go there several times for [laboratory] tests and reports.” (A 32*-*year*-*old male respondent with an anxiety disorder)*

#### Interpersonal communications

The perceived quality of interpersonal communication was measured by eight statements: (1) being greeted and asked to take a seat by care provider, (2) gentle/sympathetic behaviour of care provider, (3) privacy, (4) current health condition explained, (5) encouraged to ask questions, (6) response to the concerns/questions by care provider, (7) sufficient consultation time and (8) gentle behaviour and helpfulness of other OPD staffs. The average score for this sub-section was 7.6. The respondents were very satisfied with being greeted and asked to take a seat, gentle/sympathetic behaviour of the care provider and being provided privacy. They rated the remaining statements as satisfactory. However, during in-depth interviews, a few respondents shared their unpleasant experiences with some of those points. One respondent expressed her view about the behaviour of OPD care providers:

*“The lady doctor is not nice. She is very arrogant and often misbehaved with me. I felt afraid to ask her any question. So now I don’t go to her room any more. When I buy consultation ticket, I ask the woman to write the room number of a male doctor. The male doctors here behave nice.” (Mother of an 8*-*year*-*old male patient with a conduct disorder)*

During in-depth interviews, a few respondents shared their dissatisfaction with privacy during consultations in a particular area of the OPD meant for minor patients. One respondent stated:

*“There is only one consultation room at Shishubikash [special OPD clinic for child patients] and 3*-*5 patients are seen by the doctors at the same time. There is no environment to talk freely. Also, the doctors give very little time to the patients and just rush to finish all those waiting outside.” (Mother of an 11*-*year*-*old male patient with autism)*

Some respondents expressed their concerns in in-depth interviews about not receiving information about their mental health problem and future prospects. One respondent stated:

*“I always feel concerned about whether or when my son will recover completely. Or will he have to take medications like this his entire life? Doctors do not respond to my question. My son is also having [a] sexual problem recently. He does not have any sexual relation with his wife. I asked the doctor about this problem, whether there is any medication to solve this problem. I did not get any response to this question too. I feel worried that his wife would leave him. It is also needed for his wife.” (Mother of a 28*-*year*-*old male patient with personality disorder)*

The poorest rating in this sub-section was given for “sufficient consultation time”. This statement received dissatisfactory scores from 17 respondents and the average score was 6.3. The following quote demonstrates the disappointing experience of one respondent:

*“He is taking many medications. I brought all of those today, wanted to show, but the doctor here didn’t see those. They don’t give enough time to the patients.” (Mother of a 15*-*year*-*old male patient with mental retardation)*

#### Condition of the waiting and consultation rooms

In this sub-section, the respondents rated the condition of the waiting and consultation rooms based on eight statements: (1) cleanliness of waiting and consultation rooms, (2) enough light and ventilation, (3) comfort of waiting and consultation rooms, (4) availability of seating, (5) availability of OPD patients’ toilet, (6) cleanliness and usability of toilet, (7) availability of water/toilet paper in the toilet and (8) availability of waste bin in OPD. The average score for this sub-section was 5.8. The respondents were very satisfied only with sufficient light and ventilation. They were dissatisfied with the cleanliness and usability of toilet and availability of waste bin in the OPD. About two-thirds of the respondents gave dissatisfactory scores for these two statements and the average scores were 4.4. The rest of the statements were rated as good. The following quotes are from two respondents about their dissatisfaction:

*“We come from so far and then have to wait for hours to see a doctor. There is not even sufficient sitting arrangement at Shishubikash [special OPD clinic for child patients]. How long someone can keep standing, particularly children and elderly people?” (Grandmother of a 3*-*year*-*old male patient with autism)*

*“I don’t know when we will reach home; she is saying that she has to go to toilet. But the toilets here are so dirty. Door locks are also broken in some of those, so cannot be used.” (Daughter of a 65*-*year*-*old female patient with dementia)*

#### General quality of OPD services

In this sub-section, the respondents rated the general quality of OPD care services on the basis of nine statements: (1) attentive and patient hearing by care provider, (2) further relevant query by care provider, (3) physical examination/observation, (4) explanation about treatment/advice/possible prognosis, (5) information on possible side effects of medication, (6) information on follow up visit, (7) professional competence of care provider, (8) instructions by the pharmacist about intake of medication and (9) overall satisfaction with the quality of OPD care. The average score for this sub-section was 7.1. The respondents rated the statements regarding attentive and patient hearing by care provider, information on follow up visit, professional competence of care provider and overall satisfaction about the quality of OPD care as very good. The rest of the statements were rated as good. The following respondent indicates his confidence in the professional competence of the doctors working at this institute:

*“The services are good. People from other countries can receive treatment here too. Doctors are trained here, doctors are taught.” (Father of a 35*-*year*-*old male patient with schizophrenia)*

On the other hand, some respondents shared their unsatisfactory experiences at OPD. The quote below is from an attendant expressing her concerns about the way her patient was being treated:

*“The lady doctor doesn’t allow my son entering her room while consulting. She tells me to keep him standing outside her room. So, I enter her room alone and tell her about my son’s problem. Then she writes treatment without seeing or talking to the patient. She says that my son might attack her suddenly. So, it is not safe for her that I take my son inside her room. But, is it right that the doctor treats a patient like this?” (Mother of a 28*-*year*-*old male patient with personality disorder)*

About half of the respondents provided dissatisfactory scores for the statements about information on possible side effects of medication and instructions by the pharmacist about intake of medication, which, on average, also received dissatisfactory score and marginally satisfactory score respectively (5 and 5.6 respectively). In an in-depth interview, one respondent mentioned the extraordinary support she received from an OPD provider at NIMH. The provider shared his personal mobile phone number with her, so she could seek his advice in case of an emergency after starting the prescribed medications. On the contrary, another patient suffered from medication side effects for some days due to lack of information and instructions. The following two quotes are from these two respondents reflecting their contrasting experiences:

*“I called the doctor and said that after giving him those medications, he has become somewhat unconscious. Then the doctor advised me to stop one medication and to bring the patient here.” (Wife of a 65*-*year*-*old male patient with dementia)*

*“After taking those tablets, the child became completely tired. The way I used to lay him down, he stayed like that. The baby could not get up, could not sit. Still I continued those tablets for two weeks, as I was not told about what to do in such situation. Then I rushed to this hospital, after giving those tablets for two weeks, with the fear that what has happened to my child. Then I was advised to stop that medication.” (Grandmother of a 3*-*year*-*old male patient with autism)*

The quote below informs us about the kind of problem one respondent faced due to a lack of instructions by the pharmacist at the hospital dispensary about his medications:

*“But, one problem is that sometimes when I receive medications from this dispensary they don’t explain which one has to be taken when and how many and so on. If I ask him, then he says that it’s written in the prescription. But some names of those medications do not match with the prescription.” (Wife of a 55*-*year*-*old male patient with dementia)*

One of the respondents raised concerns about the common attitude of mental health care providers in Bangladesh that results in a hassle and bad quality of care for the patients. His remarks are as follows:

*“The doctors in our country have problems of jealousy. If I go to a neurologist, then they tell me not to go to a psychiatrist because according to them psychiatrists only prescribe sleeping pills, which doesn’t solve the real problem. If I go to a psychiatrist, then they do not want me to receive counselling from psychologists because according to them, medications cure all problems and counselling is not necessary. They speak negative[ly] about each other and do not work together for the betterment of the patients.” (25*-*year*-*old male patient with insomnia)*

### Medical equipment and human resources

#### Functionality of medical equipment

Table 3 presents the information on medical equipment at NIMH (functionality and number). Much of the medical equipment at NIMH has been non-functional for a long time. Both X-ray machines have never been installed. The only electrolyte analyser and electroencephalography (EEG) machine have been out of order since 2012, and the only magnetic resonance imaging (MRI) machine has been out of order since 2013. Some of the autoclave machines, electrocardiograph (ECG) machines, electric centrifuge machines, electroconvulsive therapy (ECT) machines, electric suction machines and refrigerators have also been out of order for several years.

**Table 3:** Number and non-functionality of NIMH medical equipment

Items	Available (total)	Out of order (n)	Not installed (n)	Duration of non-functionality
X-ray machine (300 mA)	1	–	1	Since 2012
X-ray machine (digital)	1	–	1	Since 2012
MRI machine	1	1	–	Since 2013
Autoclave	4	2	–	Since 2012
ECG machine	4	1	–	Since 2012
Centrifuge machine (electric)	5	2	–	Since 2012
Electrolyte analyser	1	1	–	Since 2012
ECT machine	2	1	–	Since 2012
EEG machine	1	1	–	Since 2012
Refrigerator	6	1	–	Since 2012
Suction machine (electric)	5	1	–	Since 2013

#### Physicians’ positions at NIMH

As shown in Table 4, from 2012 to 2016 some sanctioned (allocated) positions for physicians at NIMH were not filled, and the vacancy rate for physicians was 17.78% in 2016.

**Table 4:** Physicians’ positions at NIMH

Year	Sanctioned position	Filled position	Vacant position	Vacancy as % of sanctioned positions
2012	45	42	3	6.66
2013	43	39	4	9.3
2014	43	39	4	9.3
2015	42	37	5	11.9
2016	45	37	8	17.78

#### Physicians’ workload in the OPD

The OPD is open for services from 8 am to 2 p.m. During the 6-h time slot available for consultation, the maximum number of patients seen by one provider was 102. The average consultation time per patient on that day was 3.5 min (possible personal break time not excluded). The highest number of average patients seen in a day by one OPD physician was 42. This means that he/she could spend about 8.6 min per patient on average (personal break time not excluded). Table 5 presents the workload of OPD physicians.

**Table 5:** Workload of OPD physicians at NIMH

OPD rooms	Provider 1	Provider 2	Provider 3	Provider 4
Mean patients seen per day	23	42	37	39
Median	21	42	38	41
Maximum	101	102	88	96

## Discussion

We chose a mixed method design to not only assess respondents’ perception indicators, but also to achieve a detailed understanding of their experience and expectations. We have presented organizational limitations that we believe directly contributed to some of the negative care experiences. Thus, our study has drawn a comprehensive picture of care at NIMH in relation to the respondents’ experience and perception. A previous quantitative study on the experience and perception about the quality of maternal and new-born care in Malawi suggested that a quantitative study should be complemented by a qualitative study to unravel the complexity of experience and respondents’ perception of the quality of care [4].

Our study findings show that the respondents perceived the accessibility of OPD care, interpersonal communications as very good quality, and the general quality of OPD care as good quality. But they considered the condition of waiting and consultation rooms to be of marginally good quality. There is lack of resources at NIMH in terms of functional medical equipment and human resources resulting in limitations in health care provision.

According to the Bangladesh health system review document published in 2015, the health care providers in the public domain in Bangladesh are reluctant to provide required information to their patients. There is lack of information regarding the availability of health services, which poses obstacles to access [5]. But our respondents had a positive view regarding the access to information at NIMH. This 2015 review document also revealed that the user charges for outpatient consultations at the public health facilities are low, but patients might need to spend money for medications, laboratory tests and other medical items like syringes, dressings, or intravenous fluids [5], which is quite similar as our study findings in the NIMH. Access to care providers and affordable and acceptable health care on time are prime concerns of the patients [2]. Although various studies have reported on staff absenteeism and the existence of informal payments at public health facilities in Bangladesh [5], none of our study participants mentioned this occurring in the NIMH OPD.

As per user concerns, health service quality includes both tangible (e.g. infrastructure) and intangible (e.g. empathy) attributes [2]. In addition to good clinical performances by the doctors, patients expect care, concern and courtesy too. Polite and sympathetic provider behaviour makes the patient feel comfortable [4, 7]. Other studies conducted among OPD patients in Iran and Europe have found that a feeling of reassurance is related to a positive perception of care quality by both patients and parents [9] and patient satisfaction increases with perceived provider empathy and explanations [2, 8].

In a patients’ survey, a willingness to provide explanations was indicated as the most important criteria in choosing a provider [7]. Patients feel less worried if they are informed and explained about their condition and intervention [2, 7], which builds a positive perception about the quality of their care [4]. Many patients with a mental illness are not always able to understand or make decisions, and are fully dependent on their family. Therefore, the patients’ families should also be informed and assured about the patient’s treatment plan [7]. Communication with the patient by the provider should be performed in an understandable language and acceptable manner [2, 7]. Unfortunately, the Bangladeshi health system is not responsive or obliged to share information about the patient’s condition, treatment or prognosis with patients or their relatives [5]. A lack of training and high workload might be the reasons behind poor personal attitudes and behaviour of care providers [2]. Moreover, many mental health patients experience stigma even by health care providers [23].

Patients perceive good quality services if they are given the scope to ask questions and their questions are answered [7]. Patients also expect that providers maintain their privacy, confidentiality and dignity at every step of care provision [2, 4, 7]. Long waiting times and insufficient consultation times negatively influence patients’ perceptions of care quality [2, 7]. As per user experience, people perceive the Bangladeshi public health system as poor due to long waiting time, absenteeism, poor provider behaviour and the exclusion of some marginalized groups [5]. There is no functioning system of reward and punishment and no effective monitoring of doctor, nurse or staff performance in public health facilities in Bangladesh [5].

A clean, bright, well equipped and comfortable room creates a positive impression among patients and a subsequent positive view about the overall service quality [2, 7]. The need for maintenance of toilets and bathrooms at NIMH has been pointed out in the local health bulletin since 2013 [19, 24–26]. Our study findings indicate that no action has been taken to improve this situation.

In a WHO assessment, the public health facilities in Bangladesh were noted to have 20% constant vacancies in the allocated positions for doctors and a critical shortage of skilled human resources for mental health in particular [5]. In Bangladesh, there are 0.073 psychiatrists/100,000 population [27] compared to 12.9/100,000 in EU countries [28]. Mental health care services are almost absent at primary or secondary health facilities in Bangladesh [29]. Health facilities are poorly equipped with medical devices, instruments and supplies [5]. The lack of resources definitely has negative consequences on the service quality. Due to the absence of proper maintenance systems for medical equipment at NIMH, access to related services is hampered and causing inconvenience to the patients and their attendants. Online NIMH health bulletins [19, 24–26] published yearly by the health ministry have raised the issue of the non-functionality of x-ray machines since 2013 and the MRI since 2016 with special importance. The lack of infrastructural capacity, logistics and manpower including doctors, nurses and other support staffs, has also been mentioned in those bulletins since 2013. Presumably, no effective measure has been taken to solve those issues. Like Bangladesh, the lack of resources for mental health including infrastructure, skilled staff and medication has been documented as one of the greatest challenges for ensuring quality of care in other resource-limited countries [11, 13].

### Study strengths and limitations

We believe there are several reasons the respondents in this study may have over-rated the quality of services at NIMH. First, there may have been a bias of politeness, since straightforward criticism is not a cultural norm in Bangladesh. Respondents might have been afraid of being overheard by the NIMH care providers during their interviews and been concerned about negative consequences in their care services. Some respondents might not have been aware of international expectations of quality of care. So, they might not have been able to compare their care received with standard care. In Bangladesh, many patients and their attendants are not aware of their health rights and accept any quality of care, particularly when care is provided free of cost at a public health facility.

Due to the small sample size, the results of our quantitative survey are not representative of the whole population. A larger study would be worthwhile to assess the quality of mental health care in Bangladesh more broadly. However, there is lack of research about mental health systems in Bangladesh and our study findings do provide valuable information about this topic. The qualitative data provides additional insight about the quantitative scores provided by the respondents for quality assessment. Thus, a mixed-method design has facilitated the generation of more detailed information about the experience and perception of the NIMH OPD patients and their attendants.

Literature on various aspects of mental health care quality is scarce. Therefore, we have also cited some relevant articles on other medical care. However, we acknowledge that mental health care is unique within health care. Although some aspects of mental health care are similar to those in other medical care, quite some dissimilarities also exist; hence, those cannot be considered equivalent.

## Conclusions

The respondents perceived the services of NIMH OPD to be good quality, but their satisfaction about the condition of waiting and consultation rooms was marginal. There were particular respondent concerns indicating the need for specific improvements. There is need for increased resources for medical equipment and skilled human resources, which ultimately affect respondents’ experience and perception of quality of care. Our findings are expected to be very useful in improving the quality of mental health care services at NIMH, Bangladesh.

The perceived quality of health care cannot be used as an indicator for assessing the technical quality of care services, because patients lack knowledge about these aspects of care, thus they tend to judge the overall quality of care through their subjective perceptions and individual experiences [2, 4]. However, tangibles (physical facilities e.g. structure, building, equipment and personnel e.g. quantity and quality) and amenities (comfort of physical surroundings and attributes of the organization of service provision) influence patient confidence and trust in other aspects of services [2]. A positive perception of care eventually enhances health service utilization, treatment adherence and treatment outcomes. Therefore, it is necessary to establish a well-functioning routine feedback system for NIMH health clients to share their experiences, expectations and suggestions. We suggest that the quality of care at NIMH should be assessed every five years and based on the findings, relevant quality-improvement measures should be undertaken. We hope that by the grace of increasing high-quality and patient-satisfactory services at NIMH, mental health care coverage and outcomes will be improved greatly.

## Data Availability

Data and tools used are available from the corresponding author upon reasonable request.

## Abbreviations

NIMH: National Institute of Mental Health
OPD: outpatient department
ICD: International Classification of Diseases
WHO: World Health Organization
EEG: electroencephalography
MRI: magnetic resonance imaging
ECG: electrocardiograph
ECT: electroconvulsive therapy
